# Enhanced Visual Cortex Activation in People With Narcolepsy Type 1 During Active Sleep Resistance: An fMRI-EEG Study

**DOI:** 10.3389/fnins.2022.904820

**Published:** 2022-06-27

**Authors:** Jari K. Gool, Rolf Fronczek, Peter Bosma, Johan N. van der Meer, Ysbrand D. van der Werf, Gert Jan Lammers

**Affiliations:** ^1^Sleep-Wake Centre, Stichting Epilepsie Instellingen Netherland (SEIN), Heemstede, Netherlands; ^2^Department of Neurology, Leiden University Medical Centre, Leiden, Netherlands; ^3^Anatomy & Neurosciences, Vrije Universiteit Amsterdam, Amsterdam, Netherlands; ^4^Amsterdam Neuroscience, Mood, Anxiety, Psychosis, Sleep & Stress Program, Amsterdam, Netherlands; ^5^Department of Radiology and Nuclear Medicine, University of Amsterdam, Amsterdam, Netherlands

**Keywords:** central disorders of hypersomnolence, hypocretin, narcolepsy, magnetic resonance imaging, fMRI, sleep resistance, maintenance of wakefulness test (MWT)

## Abstract

The brain activation patterns related to sleep resistance remain to be discovered in health and disease. The maintenance of wakefulness test (MWT) is an objective neuropsychological assessment often used to assess an individual’s ability to resist sleep. It is frequently used in narcolepsy type 1, a disorder characterized by impaired sleep-wake control and the inability to resist daytime sleep. We investigated the neural correlates of active sleep resistance in 12 drug-free people with narcolepsy type 1 and 12 healthy controls. Simultaneous fMRI-EEG measurements were recorded during five cycles of two alternating conditions of active sleep resistance and waking rest. Cleaned EEG signals were used to verify wakefulness and task adherence. Pooling both subject groups, significantly higher fMRI activation when actively resisting sleep was seen in the brainstem, superior cerebellum, bilateral thalamus and visual cortices. In controls the activation clusters were generally smaller compared to patients and no significant activation was seen in the brainstem. Formal comparison between groups only found a significantly higher left primary visual cortex activation in patients during active sleep resistance. The active sleep resistance paradigm is a feasible fMRI task to study sleep resistance and induces evident arousal- and visual-related activity. Significantly higher left primary visual cortical activation in patients could be caused by an enhanced need of visual focus to resist sleep, or reflecting a more rapid descent in their level of alertness when resting.

## Introduction

The maintenance of wakefulness test (MWT) is a polysomnographic procedure to quantify daytime sleepiness and is often used to assess fitness to drive in people with excessive daytime sleepiness. During four daytime 40-min sessions at two-hour intervals, subjects are asked to resist the urge to fall asleep while sitting down in a soporific environment (Mitler et al., 1982). The underlying neural correlates of active sleep resistance largely remain to be discovered and could provide important insights in sleep-wake regulation in health and disease.

The MWT is frequently used as objective parameter in pharmacological trials and in the assessment of fitness to drive in people with narcolepsy type 1 (Littner et al., 2005); a sleep disorder characterized by excessive daytime sleepiness and cataplexy (transient episodes of muscle weakness). People with narcolepsy type 1 often have difficulty resisting sleep during repetitive tasks within a work or academic environment, or while participating in traffic (Bassetti et al., 2019). To date, little is known about the neural activation of patients’ sleep attacks and their continuous fight to remain wakeful. In this study, we quantified brain activation patterns related to sleep resistance in healthy, and troubled sleepers with narcolepsy type 1, through concurrent functional magnetic resonance imaging and electroencephalography (fMRI-EEG) recordings.

Use of simultaneous fMRI-EEG measurements is crucial for reliable sleep-wake fMRI research, as it allows for objective consciousness assessment and task adherence, while simultaneously recording whole brain activation patterns. Combining these two powerful neuroimaging techniques brings its challenges, as the EEG signal is subject to ballisto-cardiac (BCG), and MR-induced gradient and helium pump artifacts (Bullock et al., 2021). We have recently developed a unique carbon-wire loop-based (CWL) artifact correction approach that carefully filters these artifacts, while preserving true brain signals for sleep-wake scoring (van der Meer J. N. et al., 2016).

The aim of this study was to validate feasibility of the active sleep resistance paradigm through simultaneous fMRI-EEG recordings, and to assess brain activation patterns related to sleep resistance in health and disease. We hypothesize people with narcolepsy type 1 to find it more difficult to remain wakeful as compared to controls, and patients to more heavily depend on activation of the ascending reticular activating system and attention regions when actively resisting sleep.

## Materials and Methods

### Participants

Twelve healthy adults and twelve age and sex group-matched people with narcolepsy type 1 were included. People with narcolepsy type 1 were diagnosed according to the 3rd edition of the International Classification of Sleep Disorders (ICSD-3) (American Academy of Sleep Medicine [AASM], 2014). All participants had to be 18-65 years old, right-handed and have normal or corrected-to-normal visual acuity. Patients were medication-free for at least two weeks before MRI acquisition. Exclusion criteria were a serious comorbidity, contraindications for MRI and macroscopic structural brain abnormalities. Subjects were asked to refrain from caffeine-containing substances 24 h prior to measurement. The Dutch National Adult Reading Test (Schmand et al., 1991) was administered to assess intelligence and the Epworth Sleepiness Scale (ESS) (Johns, 1991) was used to measure daytime sleepiness. Written informed consent was provided beforehand. The study was approved by the Medical Ethical Committee of Leiden University Medical Center (LUMC), Netherlands.

### Active Sleep Resistance Paradigm

The active sleep resistance paradigm lasted 5 min in total and consisted of five cycles of alternating 30-s blocks of active sleep resistance with eyes open and waking rest with eyes closed. The scanner room light was dimmed and transitions between task conditions were indicated through brief presentation of a bright white screen that was noticeable through closed eyelids. EOG and alpha activity fluctuations were checked to assess task adherence through opening and closing of the eyes.

### Electroencephalography Acquisition and Processing

High-density EEG recordings were acquired and pre-processed in accordance with van der Meer J. et al. (2016), van der Meer J. N. et al. (2016). In brief, five twisted CWLs were symmetrically sewn through the 256 electrode EEG cap (MicroCel Geodesic Sensor Net, Electrical Geodesics, Inc.) that captured BCG and other movement-related artifacts. The helium pump was temporarily switched off to prevent associated artifacts.

EEG data were pre-processed in MATLAB using EEGlab. We removed the MRI artifacts from the EEG recordings using the Bergen EEG-fMRI toolbox (Moosmann et al., 2009). CWL and EEG signals were downsampled to 500 Hz and band-pass filtered between 0 and 100 Hz using the EEGlab CWL plugin (van der Meer J. et al., 2016). To remove BCG and other movement-related artifacts, the signals from the five CWLs were hereafter combined, aligned with the EEG signals and subtracted from the individual EEG electrode signals through sliding Hanning window regression (six second windows). This final step eliminated the non-brain signals from the EEG signals. Cleaned EEG recordings were used for sleep scoring according to AASM criteria using 30 s epochs; note, though, that no EMG measurements were obtained.

### Functional MRI Acquisition and Processing

Whole-brain T2*-weighted and T1-weighted scans were acquired with the same parameters as in Gool et al. (2020b). T2*-weighted MRI data were acquired using a gradient-echo planar imaging (EPI) sequence (38 slices with a 0.25 mm gap; repetition time [TR] = 2250 ms; echo time [TE] = 29.94 ms; field of view [FOV] 200 mm × 200 mm × 104.25 mm; matrix size 80 × 80; flip angle = 80°; 2.5 mm × 2.5 mm × 2.5 mm voxel size). T1-weighted MR images were acquired (220 slices; TR 8.2 ms; TE 3.8 ms; inversion time 670.4 ms; FOV 240 mm × 240 mm × 220 mm; matrix size 240 × 240; flip angle 8°; 1 mm × 1 mm × 1 mm voxel size). The fMRI images were pre-processed and analyzed using FMRIB’s Software Library (FSL FEAT, version 6.0.4). Scans were motion corrected using MCFLIRT, brain extracted, normalized, filtered using a 100 s high-pass filter, smoothed with a 5 mm full-width at half maximum (FWHM) Gaussian kernel and coregistered to the corresponding skull-stripped T1 image. FILM prewhitening was implemented as a non-parametric estimation of each voxel’s time series autocorrelation, a temporal derivative was added to account for hemodynamic response function deviations and the derived motion parameters were included as nuisance regressors in the general linear model.

First-level within-subject effects were assessed using the mean BOLD response of the sleep resistance blocks in comparison to mean activation of the wakeful rest blocks. The transition periods between task conditions, possible sleep epochs, and possible delays in following task instructions as checked through EOG monitoring, were excluded in first-level analyses.

The second-level main task fMRI effects were determined per group using one-sample *t*-tests. For group comparisons, two-sample t-tests were used. The main task effect analyses were performed in the entire sample and controlled for type I errors using family-wise error (FWE) correction. Cluster-correction was implemented for the group comparison. Two *post hoc* analyses were performed for people with narcolepsy to test whether between group differences were related to patient-specific markers. ESS scores and disease duration – measured as duration since EDS onset – were, respectively, added as covariates of interest in second-level analyses just including people with narcolepsy. A *p* < 0.05 threshold and minimum significant clusters size of 20 voxels were used. Locations of significant clusters were determined using the AAL and Brodmann + atlases in WFU PickAtlas (part of SPM12).

## Results

Both groups were comparable in age, sex and IQ distributions (Table 1). Patients had typical narcolepsy type 1 characteristics, were all HLA DQB1*0602 positive and had significantly higher ESS scores than controls. One epoch during the waking rest condition was scored as NREM1 sleep in one patient and this period was excluded from the fMRI analyses.

**TABLE 1:** Characteristics of the study population.

	Patients (*n* = 12)	Healthy controls (*n* = 12)	*P*-value
Male:female (N:N)	8:4	8:4	1.000
Age (years, mean, SD)	33.25 (10.50)	32.75 (13.16)	0.919
IQ score (mean, SD)	110.58 (10.73)	111.30 (8.25)	0.865
Age of onset EDS (years, mean, SD)	19.42 (9.15)	−	
EDS duration (years, median, IQR)	10.00 (6.00-25.25)	−	
Cataplexy presence	9/12	−	
Cataplexy and/or hypocretin deficient (N,%)	12/12	−	
HLA DQB1*0602 presence (N,%)	12/12	−	
ESS score (mean, SD)	10.08 (3.00)	2.67 (1.87)	<0.001
MSLT:			
- Sleep latency (minutes, mean, SD)	4.62 (3.64)	−	
- SOREM periods (mean, SD)	2.58 (1.57)	−	

*EDS, excessive daytime sleepiness; HLA, human leukocyte antigen; ESS, Epworth sleepiness scale; MSLT, multiple sleep latency test; SOREM, sleep-onset rapid eye movement.*

### Main Task Effect

**FIGURE 1:**
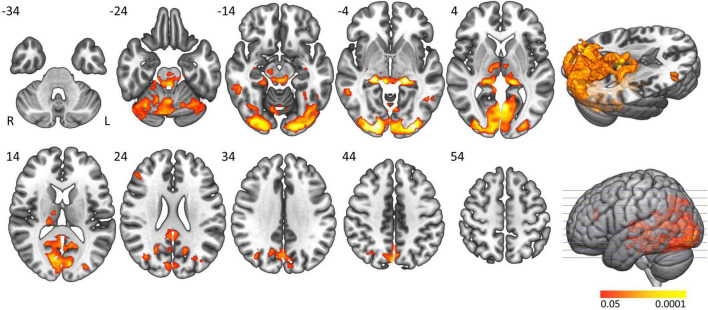
Main task effect activation clusters. Axial slices displaying significantly activated voxels in the “active sleep resistance > waking rest” contrast over all subjects. Analyses were cluster-corrected (*p* < 0.05), masked for gray matter, and a minimum cluster size > 20 voxels was used.

The ‘active sleep resistance > waking rest’ contrast elicited widespread and almost symmetrical significant BOLD activation in regions related to consciousness (brainstem including bilateral locus coeruleus and thalamus), attention (cerebellum, precuneus and thalamus) and wake-related visual cognitive activation (thalamus, occipital, and inferior and middle temporal lobe) (Table 2 and Figure 1). The ‘waking rest > active sleep resistance’ contrast showed smaller significant activation clusters (Table 2).

**TABLE 2:** Main task effect activation clusters.

Contrast	Anatomical regions (AAL atlas)	Anatomical regions (Brodmann + atlas)	Cluster size	Peak *Z*-value	*P*-value (Cluster)	x, y, z
Active sleep resistance > waking rest	Occipital: Calcarine gyri, lingual gyri, inferior, middle and superior occipital gyri, cuneus (bilateral) Cerebellum: Cerebellum 3-6 and vermis 1, 2, 5-7 (bilateral), crus cerebelli 1 (R) Parietal: Precuneus (bilateral) Temporal: Fusiform gyri, parahippocampal gyri (bilateral) Subcortical: Thalamus, hippocampus (bilateral) Posterior cingulate cortex (bilateral)	Bilateral area 18, 17, 19, 30, 7, 23, 31, 29, 27, 37, 35, 28 Bilateral thalami (pulvinar, mediodorsal, ventral and lateral posterior nuclei) Bilateral globus pallidus Midbrain (right substantia nigra) Pons (bilateral locus coeruleus)	12158	6.29	<0.0005	−20, −30, −2
	Middle and inferior temporal gyri (R)	Area 20 (R)	193	4.23	<0.0005	56, −44, −4
	Middle and superior occipital gyri, angular gyrus (R)	Area 19 (R)	167	4.21	0.001	36, −68, 42
	Inferior (pars triangularis) and middle frontal gyri (R)	Area 46 (R)	93	4.19	0.015	44, 32, 18
	Inferior temporal gyrus (L)		75	4.52	0.039	−48, −54, −6
Waking rest > active sleep resistance	Middle temporal gyrus (R)	Area 37 (R)	103	4.77	0.009	56, −66, 0
	Fusiform and inferior temporal gyri (R)	Area 20 (R)	86	4.44	0.022	42, −18, −30

*Overview of significant main task activation clusters. Analyses were cluster-corrected (p < 0.05), masked for gray matter, and a minimum cluster size > 20 voxels was used.*

### Group Differences

**FIGURE 2:**
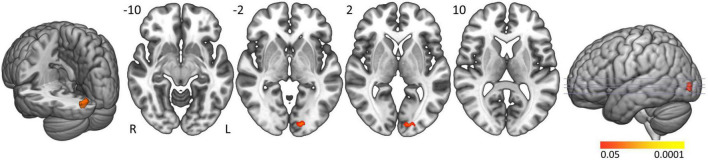
Active sleep resistance group differences. Axial slices displaying significant activation clusters that were significantly more activated in people with narcolepsy type 1 than in controls during active sleep resistance. Analyses were cluster-corrected (*p* < 0.05), masked for gray matter, and a minimum cluster size > 20 voxels was used.

In controls the activation clusters in general were smaller compared to people with narcolepsy type 1 and no significant activation was seen in the brainstem. Formal comparison between groups elicited significantly greater activation in people with narcolepsy type 1 in the left calcarine and lingual gyri (Brodmann area 17; cluster size: 43 voxels; peak z-value: 3.88; cluster-corrected *p*-value = 0.0463; Z-max MNI coordinates: −42, −4, 36) when actively resisting sleep (Figure 2). No clusters were found with significantly more activation in controls compared to patients. Neither of the *post hoc* analyses with ESS scores and EDS duration as covariates of interest revealed significant correlations between these clinical markers and the observed group difference in the left calcarine and lingual gyri.

## Discussion

The active sleep resistance paradigm is a feasible MRI task that elicits strong BOLD activation during the active sleep resistance condition in brain regions related to consciousness regulation, attention maintenance and sensory information processing. Within the active sleep resistance blocks patients showed significantly more activation in the left lingual and calcarine gyri compared to healthy controls. No significant correlation was found with ESS scores and EDS duration for patients in these regions, suggesting that the observed group differences are likely related to having narcolepsy and not the disease severity or duration.

No studies have yet investigated the neural activation pattern of active sleep resistance using MRI. The widespread visual activation that is induced during sleep resistance likely results from a combination of active sleep resistance and open vs. closed eyes. Research by Qin et al. (2012) with similar power and block-design setup suggests that our task activation clusters are roughly double the size of what could be expected from just using open vs. closed eyes conditions. This implies that our measured effect, to a considerable extent, is attributable to active sleep resistance. Previous EEG research in psychiatric disorders (Sander et al., 2015) and during the MWT in multiple sleep disorders (including narcolepsy type 1) revealed that slowing of occipital activity is related to microsleep occurrence (Hertig-Godeschalk et al., 2020). The increased occipital activation during the task likely reflects the opposite process, where participants actively recruit their occipital mental capacities to remain wakeful.

The increased activation in people with narcolepsy type 1 in the visual cortex could be explained by an enhanced need of visual focus to resist sleep, or lower activation of this area during waking rest in patients, reflecting a more rapid descent in the alertness level of individuals with narcolepsy type 1. Patients generally reported that they had to intensely focus on all available stimuli during the sleep resistance condition. Similar patterns were seen within the same sample when performing the sustained attention to response task (SART). In this study, patients already had to utilize more cognitive effort to maintain vigilant at the start of the task, compared to healthy controls that benefited more from their cognitive reserves as the task progressed (Gool et al., 2020b). The between-group differences in the present study are, however, subtler than we hypothesized. A possible explanation could be that patients already had to utilize some of their ability to resist sleep in the waking rest condition, albeit to a lesser extent than in the sleep resistance condition. Larger participant groups and/or longer task blocks would allow to better grasp the neural substrate of active sleep resistance in both healthy and troubled sleepers.

Eye opening and fading of alpha activity generally started a few seconds after instructions of active sleep resistance were presented. More widely known as the Berger effect, this phenomenon of alpha frequency blockade when opening ones’ eyes (Henning et al., 2006) was used to review task adherence in the present study. The delay, however, differed per subject and was typically longer for patients (Supplementary Material 1). In combination with the presence of sleep bouts in patients this emphasizes the importance of including simultaneous EEG measurements when conducting fMRI measurements in central disorders of hypersomnolence. Objective monitoring of vigilance has been sporadically included in other studies, but is necessary in this sleepy population to verify task adherence and continuous wake during task-based and resting-state fMRI (Gool et al., 2020a).

Active sleep resistance induced activation within consciousness, attention regulation and visual cognitive processing-related regions. The increased visual cognitive activation in people with narcolepsy type 1 may reflect their continuous battle to stay awake during the task that would underlie their sleep attacks in everyday life.

## Data Availability Statement

The raw data supporting the conclusions of this article will be made available by the authors, without undue reservation.

## Ethics Statement

The studies involving human participants were reviewed and approved by Medical Ethical Committee of Leiden University Medical Center (LUMC), Leiden, Netherlands. The patients/participants provided their written informed consent to participate in this study.

## Author Contributions

JG: data analysis, interpretation of results, and manuscript writing. RF: conceptualization, interpretation of results, and manuscript reviewing. PB: data analysis. JM: conceptualization, data collection, data analysis, and manuscript reviewing. YW: conceptualization, data collection, interpretation of results, and manuscript reviewing. GL: conceptualization, interpretation of results, and manuscript reviewing. All authors contributed to the article and approved the submitted version.

## Conflict of Interest

The authors declare that the research was conducted in the absence of any commercial or financial relationships that could be construed as a potential conflict of interest.

## Publisher’s Note

All claims expressed in this article are solely those of the authors and do not necessarily represent those of their affiliated organizations, or those of the publisher, the editors and the reviewers. Any product that may be evaluated in this article, or claim that may be made by its manufacturer, is not guaranteed or endorsed by the publisher.
