# Supplementary material for: Enhanced Visual Cortex Activation in People With Narcolepsy Type 1 During Active Sleep Resistance: An fMRI-EEG Study
Source: Front Neurosci. 2022 Jun 27;16:904820. doi: 10.3389/fnins.2022.904820 (PMC9271668; doi:10.3389/fnins.2022.904820)
Supplement: Supplementary file 1 [file Data_Sheet_1.docx]

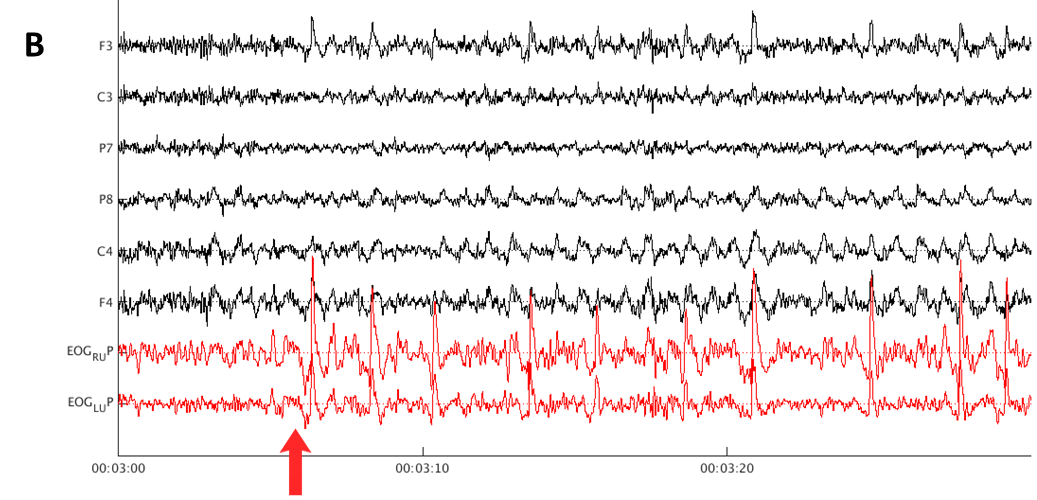

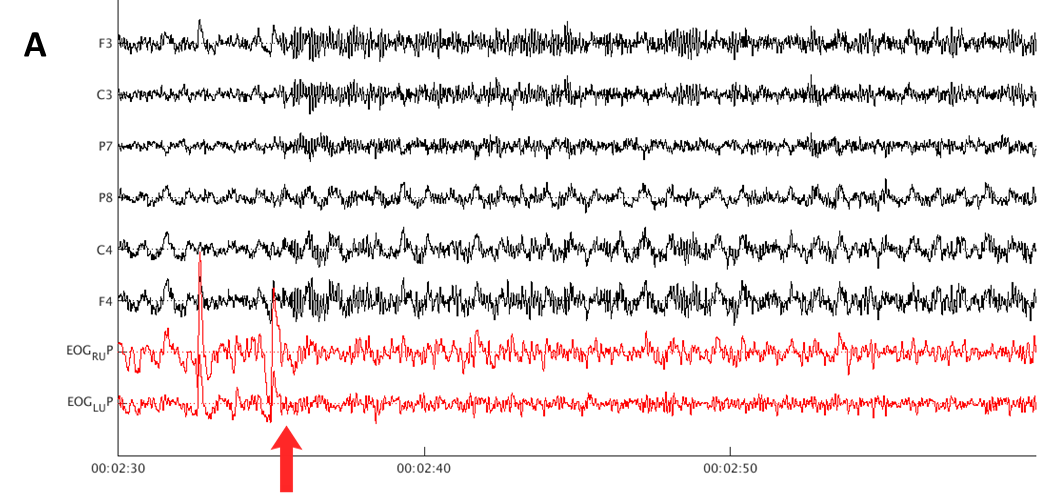
Supplementary Material

**Supplementary figure 1.** Representative electroencephalography (EEG) and electrooculography (EOG) traces of one patient with narcolepsy type 1 to illustrate the delay in task adherence when switching between the two conditions. Delay was typically shorter in healthy controls. **A.** At 2:30 (beginning of this epoch), the instructions were presented to close the eyes for waking rest. The patient followed the instructions 5.5 seconds later (red arrow), after which alpha activity became dominant in the EEG traces with fading oculomotor activity in the EOG. **B.** At 3:00 (beginning of this epoch), the instructions were presented to open the eyes and actively resist sleep. The patient followed the instructions 6 seconds later (red arrow), after which alpha activity faded in the EEG with clear oculomotor activity in the EOG.
